# Regulation of PP2A by Sphingolipid Metabolism and Signaling

**DOI:** 10.3389/fonc.2014.00388

**Published:** 2015-01-15

**Authors:** Joshua Oaks, Besim Ogretmen

**Affiliations:** ^1^Department of Biochemistry and Molecular Biology, Hollings Cancer Center, Medical University of South Carolina, Charleston, SC, USA

**Keywords:** ceramide, sphingolipids, FTY720, PP2A, tumor suppression

## Abstract

Protein phosphatase 2A (PP2A) is a serine/threonine phosphatase that is a primary regulator of cellular proliferation through targeting of proliferative kinases, cell cycle regulators, and apoptosis inhibitors. It is through the regulation of these regulatory elements that gives PP2A tumor suppressor functions. In addition to mutations on the regulatory subunits, the phosphatase/tumor suppressing activity of PP2A is also inhibited in several cancer types due to overexpression or modification of the endogenous PP2A inhibitors such as SET/I2PP2A. This review focuses on the current literature regarding the interactions between the lipid signaling molecules, selectively sphingolipids, and the PP2A inhibitor SET for the regulation of PP2A, and the therapeutic potential of sphingolipids as PP2A activators for tumor suppression via targeting SET oncoprotein.

## Introduction

Protein phosphatase 2A (PP2A) is a serine/threonine phosphatase, which serves as a regulator of cell death and/or cell division, functions performed by targeting kinases and other effectors of these processes including Bcl-2 and the Aurora kinases (1–3). In non-malignant cells, PP2A restrains cell division to occur only when necessary stimuli is present, and induces cell death by triggering extrinsic or intrinsic death signals.

**Figure 1:**
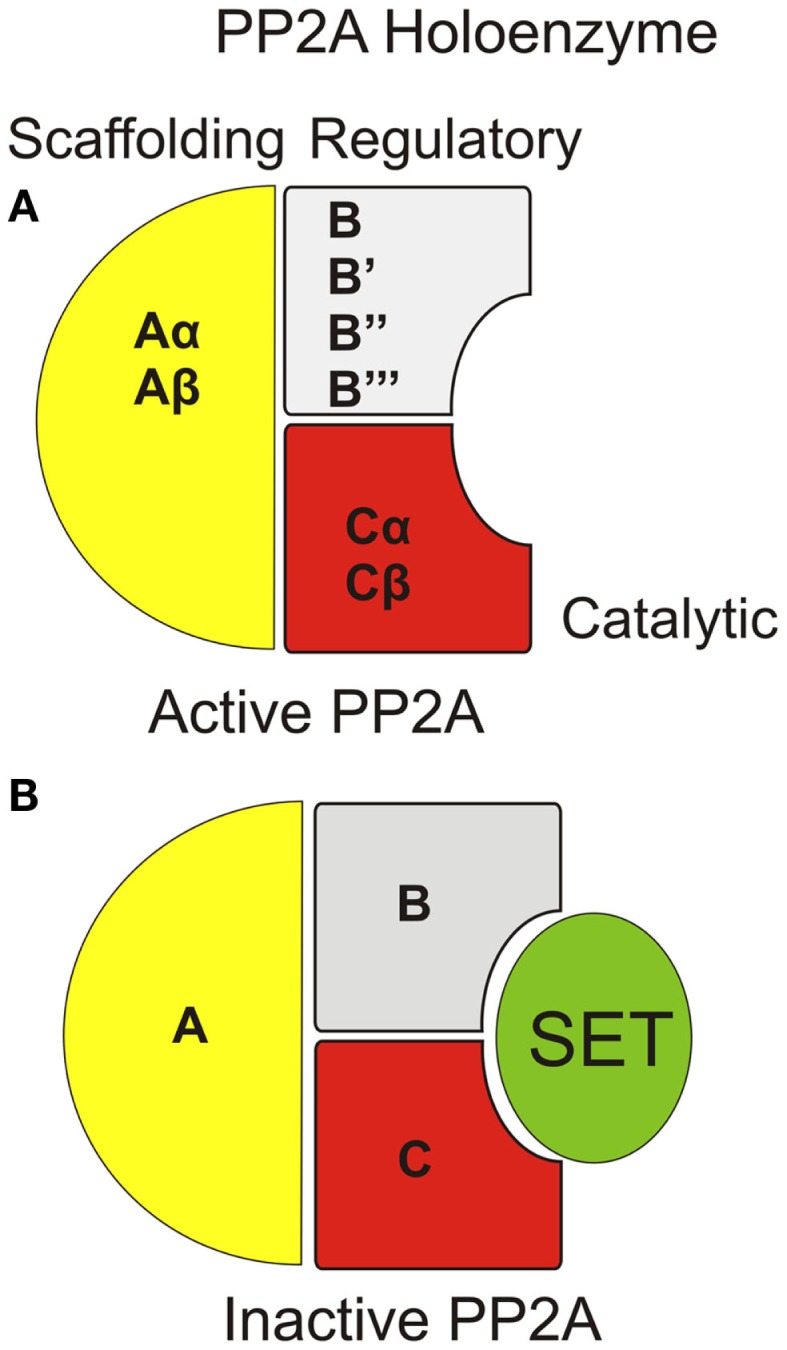
**Organization of PP2A holoenzyme with/without SET/I2PP2A interaction**. **(A)** The PP2A holoenzyme consists of a scaffolding “A,” regulatory “B,” and catalytic “C” subunit. Each subunit type can include any one of multiple species for each subunit. **(B)** SET inhibits PP2A activity through direct interaction within the catalytic domain of PP2A.

Structurally, PP2A is a holoenzyme, which typically contains a catalytic “C” subunit, coupled with an “A” scaffolding subunit, and a regulatory “B” subunit (Figure 1A) (4). Some form of PP2A holoenzyme is found in all tissues with variations of the subunit isoforms and/or post-translational modification of the subunits accounting for tissue specificity, subcellular distribution, and substrate selection. In this article, we focused on one of the major cancer-related PP2A inhibitors, SET, and its regulation by sphingolipids, primarily ceramide for PP2A reactivation and tumor suppression.

## PP2A as a Tumor Suppressor

Protein phosphatase 2A was first identified as a tumor suppressor when it was discovered that the known carcinogen okadaic acid is a specific PP2A inhibitor (5–7). Similarly, the transforming (oncogenic) simian vacuolating virus 40 (SV40) small T-antigen is believed to be tumorigenic due to PP2A inhibition (8). Likewise, PP2A activity was inhibited in a variety of cancers including chronic myeloid leukemia (CML), acute myeloid leukemia (AML), polycythemia vera (PV), and lung cancer (9–12). Importantly, reactivation of PP2A through genetic or pharmacologic means induces cell death in various cancer cells in culture and in animal models.

## Biological Inhibitors of PP2A

Protein phosphatase 2A can be regulated through changes in phosphatase activity as well as changes in substrate specificity, the latter of which is primarily controlled by the regulatory B subunits. The B subunits are often tissue-specific and mediate subcellular localization of the holoenzyme. The catalytic activity is controlled by post-translational modification of the C-subunit and through endogenous inhibitory proteins. Although the specific interplay between each of these inhibitory proteins and the PP2A holoenzyme is not fully understood, they are typically found together in a complex suggesting a role for direct inhibition.

## I1PP2A

One of these endogenous inhibitors is Inhibitor 1 of PP2A (I1PP2A, also known as acidic leucine-rich nuclear phosphoprotein 32 A or ANP32A) (13). I1PP2A (ANP32A) is a sphingosine/dimethylsphingosine-sensitive PP2A inhibitor that regulates PP2A function in human umbilical vein endothelia cells (HUVEC) (14). I1PP2A also functions as part of the histone acetyltransferase regulatory INHAT complex. Another member of the ANP32 family, ANP32e (formerly cerebellar developmental-regulated protein 1, CPD1), is a PP2A inhibitor and also plays a role in neuronal development (15, 16).

## CIP2A

Cancerous Inhibitor of PP2A (CIP2A) was first described as a fusion protein detected in a leukemia patient (17), which is an endogenous PP2A inhibitor that has been shown to have both proto-oncogenic and prognostic value in cancer. CIP2A is overexpressed in several different cancer types including head and neck squamous cell carcinoma (HNSCC), colon-cancer and CML (18), a subset of myleodysplasic syndromes (19), and osteosarcoma (20) as well as others (21). CIP2A also serves as a prognostic marker in cutaneous malignant melanoma (CMM) (22), cholangiocarcinoma (23), pancreatic ductal adenocarcinoma (PDA) (24), aggressive astrocytoma (25), and others (26, 27). Physiologically, CIP2A is particularly important for shielding c-Myc from dephosphorylation by PP2A, leading to protection of c-Myc from proteasomal degradation (28).

## SET/I2PP2A

Inhibitor 2 of PP2A (I2PP2A/SET also known as TAF-1β) is an endogenous PP2A inhibitor and a well-described proto-oncoprotein. Although the specific mechanism by which SET inhibits PP2A is believed to be through direct binding to the catalytic domain of PP2A (Figure 1B), the details of this interaction have yet to described. Similar to CIP2A, SET plays a role in a variety of cancers (29, 30) and was originally discovered as a chimeric protein (31). A fusion protein consisting of the entire SET protein sequence coupled with the carboxyl-terminal end of the CAN nuclear pore protein was found in a single acute lymphocytic leukemia patient sample and postulated to be an oncogene (32, 33). Mechanistically, SET inhibits PP2A in cell-free assays at low (nanometer) concentrations while not inhibiting related phosphatases (34). Since its discovery, SET has been found to be involved in a variety of cancers, primarily as an inhibitor of PP2A. Specifically, there are three described mechanisms by which SET has been shown to become an active PP2A inhibitor: (i) overexpression of SET is detected in several leukemias, Wilms tumors (9, 29), epithelial ovarian cancer (35), prostate cancer (36), and lung cancer (12); (ii) altered phosphorylation of SET was observed in prostate cancer, PV, Alzheimer’s disease, and in activated T-cells with the suspected kinases defined as PKC, casein kinase II, and PKD, respectively. PI3K is also suspected of phosphorylating SET for activation (11, 36–39); and (iii) changes in endogenous ceramide (an inhibitor of SET, discussed below) are also common in several cancers, reviewed in Ref. (40). Independent of PP2A inhibitory function, SET also functions to regulate gene expression through alteration of histone acetylation (41, 42).

Several of the domains of the SET protein have been attributed to specific functions. An internal span close to the N-terminus (residues 36–124) has been found to be critical for PP2A inhibitory function (43). Conversely, the C-terminal end is responsible for histone binding (44). Additional published data demonstrate that both the N- and C-terminus segments of granzyme A (45) cleaved SET at N175 to bind and inhibit PP2A (46). The coiled-coil domain (E25-Q65) is critical for SET dimerization, which modulates some, but not all, of the nuclear functions of SET. Dimerization is not necessary for SET to inhibit PP2A (47). Interestingly, the structural details of specific interactions between SET and the PP2A holoenzyme with specific A, B, and C subunits with tumor suppressor functions have not yet been described.

## SET Regulation in Human Cancers

Overexpression of SET is found in CML, Wilms tumors, and in lung cancers. In CML, an increase in heterogeneous nuclear ribonucleoprotein A1 (hnRNP A1) leads to an enhanced mRNA stability, which produces an increase in SET protein levels (9). Knock-down of SET in both CML cell lines as well as CML CD34^+^ primary patient samples restored PP2A activity indicating a causal role for SET in PP2A inhibition in these cells. Additionally, CML cells expressing SET knock-down had reduced BCR/ABL1 phosphorylation (activity) as well as reduced clonogenic potential. SET was also found to be overexpressed in lung tumors when compared to normal lung tissue with an accompanying increase in PP2A phosphorylation (inhibition) (12). Knock-down of SET in A549 lung cancer cells produced an increase in PP2A activity and a reduction in tumor proliferation *in situ* and in SCID mice. SET was found overexpressed in B-CLL cells as well as in the immortal, non-CLL B-cell cell lines, Raji and Ramos (48). Reactivation of PP2A through SET knock-down using shRNAs or a SET-targeting peptide induced cytotoxicity in both Raji and Ramos cells (48).

## Targeting SET by Sphingolipids for Tumor Suppression

**Figure 2:**
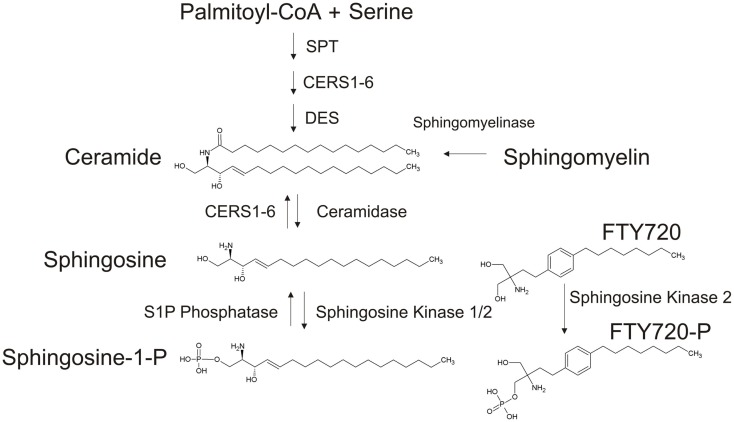
**Generation of ceramide and FTY720-P**. Ceramide can be generated through *de novo* synthesis originating with palmitoyl-CoA and serine, though the hydrolysis of sphingomyelin or through the “salvage pathway” via the acetylation of sphingosine. FTY720, a sphingosine (myriocin) analog, is phosphorylated by SphK (mainly by Sphk2 in the nucleus).

Ceramides are a class of sphingolipids consisting of a sphingosine backbone, which is N-acylated and joined to a fatty acid moiety (Figure 2). Ceramide can be generated *de novo* through a series of steps starting with serine palmitoyltransferase (SPT), which uses acetyl-CoA and serine to form 3-keto-dihydrosphingosine. This product is desaturated by dihydrosphingosine desaturase (DES) to produce dihydrosphingosine, which is the acylated by ceramide synthase (CerS1-6) to form ceramide with the desaturation reaction by dihydroceramide desaturase. Alternative pathways of synthesis of ceramide are through the breakdown of sphingomyelin by sphingomyelinases or originating directly from sphingosine produced by the dephosphorylation of sphingosine-1-phosphate (S1P) via the salvage pathway involving CerS function (Figure 2).

Ceramide was shown to activate PP2A (49–51), as well as the related phosphatase PP2C (52), without any mechanistic information. Recent data provided a unique mechanism for ceramide-mediated PP2A activation, revealing that ceramide directly binds SET, relieving PP2A from SET, increasing PP2A activity, leading to tumor suppression, consistent with anti-proliferative roles of ceramide (48–52).

FTY720 (fingolimod, Gilenya, Novartis) is a synthetic sphingosine (myriocin) analog currently approved for the treatment of multiple sclerosis. Originally designed as an immunosuppressant, FTY720 is given orally as a pro-drug. Once internalized within a target cell, FTY720 is mainly phosphorylated by sphingosine kinase 2 (SphK2), producing FTY720-P. Following phosphorylation, FTY720-P is exported out of the cell, where it functions in an autocrine/paracrine manner by binding to the sphingosine-1-phosphate receptor 1 (S1PR1). Following binding to S1PR (except S1PR2), the receptor is internalized and degraded, leading to immune suppression. Thus FTY720-P is a functional antagonist for S1PR1. FTY720 was found to activate PP2A by Nagahara et al. in HL-60RG and Jurkat cells (53). As a sphingosine analog, FTY720 retains many of the functional characteristics of natural sphingolipids, but it has a good bioavailability and a protracted half-life (54).

## Regulation of SET/PP2A Interaction by Ceramide and FTY720

While SET can be regulated through expression levels or phosphorylation changes, the inhibitory properties of SET are also controlled through sphingolipid signaling. While sphingolipids have been shown to bind another PP2A inhibitor, I1PP2A/ANP32A, SET is unique in that it contains a binding “pocket,” which affords high affinity and substrate selectivity for ceramide.

Ceramide was described as a PP2A activator in 1993 (49) and, although there has been evidence of an interaction between PP2Ac and ceramide (55), an alternative mechanism by which ceramide activates PP2A is likely through direct binding to SET.

In fact, SET was found to be a direct target of ceramide by Mukhopadhyay et al. by employing biotin-labeled C_6_-ceramide to pull-down ceramide associated proteins found in A549 lung carcinoma cell extracts using avidin-conjugated columns (56). The prominent protein found through SDS-PAGE was excised and identified though LC/MS as SET. Further examination helped identify that different species of ceramide had vastly different binding affinities for SET. First, using recombinant SET protein, our laboratory found that only the natural isomer, D-e-C_6_-ceramide, and not L-e-C_6_-ceramide binds SET. Additionally, *de novo*-generated biotin-labeled C_18_-ceramide, but not C_16_-ceramide, bound to SET following an avidin pull-down. Moreover, purified trimeric PP2A supplemented with recombinant SET was activated by the addition of water-soluble pyridinium (Pyr) D-e-C_6_-ceramide, while its isomers Pyr-dihydro-C_6_-ceramide, Pyr-L-e-C_6_-ceramide, Pyr-D-theo-C_6_-ceramide, or Pyr-D-theo-C_6_-ceramide produced no PP2A activation. Taken together, these data indicate that ceramide functions as a PP2A activator through, at least in part, the binding to SET.

This attribute of ceramide selectivity for SET binding led to the construction of a molecular model wherein ceramide could be docked and specific interactions were predicted. Using computational modeling, a hydrophobic pocket within the SET protein was identified as the expected site for ceramide binding. This pocket includes the residue K209 on helix 7, which was predicted through *in silico* modeling to be significant for ceramide binding. When mutated to K209D (replacing the positively charged R-group of lysine with the negatively charged group of aspartate), the ability of ceramide to bind SET was decreased by >75%. Moreover, ceramide-mediated PP2A activation induced proteasomal degradation of c-Myc, and tumor suppression (56).

Additional work, which demonstrates the binding of ceramide to SET, expanded on the interactions between ceramide and SET but also ascertained the mechanism of action of the PP2A activator/synthetic sphingolipid analog FTY720 (12). Data indicated preferential binding of C_18_-ceramide over C_16_-ceramide to SET. Indeed, C_18_-ceramide had the highest affinity for wild-type SET, although lesser affinity ceramides were also found to bind SET including C_20_-, C_22_-, C_26_-ceramides, and minute binding of C_24_-ceramide. Moreover, in addition to the K209 residue (56), Y122 was suspected to interact with K209 via a hydrophobic/ionic interaction. The Y122C mutant of SET was employed to determine the potential role of opening the “gate” produced through the K209/Y122 interaction with ceramide. The Y122C mutant bound to all ceramides. More importantly, by using a SET mutant, which is retained within the endoplasmic reticulum (ER) rather than the nucleus, the ER-targeted SET demonstrated high affinity for C_16_- rather than C_18_-ceramide despite the higher concentration of C_16_-ceramide in both compartments (12).

When compared to normal lung tissue, an increase in SET protein levels as well as a corresponding decrease in C_18_-ceramide were detected, indicating a two-pronged reduction in PP2A activity through both an increase in SET and a decrease in the SET inhibitor C_18_-ceramide. Moreover, mRNA levels of ceramide synthase 1 (CerS1), which is responsible for the generation of C_18_-ceramide were decreased in lung tumor tissue compared to normal lung tissue. Interestingly, the mRNA of CerS6 and its product, C_16_-ceramide were both increased in lung tumor tissue (12).

## FTY720 Reactivates PP2A

The structural and functional similarities of the synthetic sphingolipid analog FTY720 (myriocin analog) suggest that it too, may be a ligand for SET. *In silico*, molecular docking experiments indicated that the binding of FTY720, but not FTY720-P, to SET is favorable and employs the same K209 residue as ceramide. Indeed, using a combination of LC/MS, surface plasma resonance (SPR) and biotin-tagged FTY720 pull-downs FTY720, but not immunosuppressive FTY720-P, was found to bind directly to SET (12). Similar to the findings with ceramide, the K209D mutant SET did not bind FTY720 with the high affinity when compared to wild-type SET (12). These studies suggested that FTY720 and not immune suppressive FTY720-P exerts anti-cancer activities against various cancer types. Interestingly, FTY720-P was shown to inhibit colitis-mediated colon-cancer growth/proliferation (57). However, whether PP2A reactivation was involved in the suppression of colitis-mediated colon tumor growth/proliferation by FTY720-P, which appeared to be phosphorylated by tumor SphK1 in SphK2−/− mice, has not been examined.

FTY720 has favorable pharmacokinetic properties compared to natural ceramides, therefore Saddoughi et al. used FTY720 as a therapeutic drug for the treatment of A549 lung cancer cell-derived tumors. Interestingly, both FTY720 and SET knock-down prevented the growth of A549 *in situ* and *in vivo*. Surprisingly, through the use of caspase 3/7 knockout MEFs and the caspase inhibitor Z-VAD, FTY720-induced cell death was found to be caspase-independent (12). Further experiments using receptor-interacting serine/threonine-protein kinase 1 (RIP1) knockout cells, siRNA, or chemical inhibitor of RIPK1 (necrostatin) helped determine the cell death induced by FTY720 in A549 cells to be a caspase-independent, RIP1-dependent programed necrosis known as necroptosis (12).

Prior to the quantitation of SET levels in CLL by Christensen et al. (48), Liu et al. found therapeutic potential in administering FTY720 to primary CD19^+^ lymphocytes from B-CLL patients (58). Likewise, several cell lines were found to be sensitive to FTY720; MEC-1 B-CLL derived cells, Ramos and Raji cells (described above), and the acute lymphoblastic leukemia cell lines 697 and RS4;11. While SET was not examined in this study, PP2A was found to be activated by FTY720 and re-inhibition of PP2A with okadaic acid rescued cells from FTY720-mediated cytotoxicity. While the cytotoxic effects of FTY720 were found to be PP2A-dependent, the primary receptor involved in FTY720-induced immunosuppression, the S1PR1, was found to be not involved (48).

Similarly, Oaks et al. found that targeting SET with FTY720 was able to selectively kill hematopoietic progenitor cells expressing the oncogenic Jak2^V617F^ tyrosine kinase (11). Again, FTY720-P was found to be ineffective in activating PP2A. While cells expressing Jak2^V617F^ did not exhibit increased SET expression, serine phosphorylation of SET was found to require Jak2 activity. Indeed, phosphorylation of SET on the serine 9 residue was necessary for complete PP2A inhibition, consistent with data obtained in other models (37).

## Ceramide and FTY720-Mediated PP2A Activation Leads to Tumor Suppression

Mukhopadhyay et al. have found that synthetic C_6_-ceramide is a viable treatment for prostate cancer cells, which overexpress the SET protein (59). When compared to normal prostate epithelium, the androgen-resistant prostate cancer cell lines PC-3 and DU145, as well as the androgen-sensitive LNCaP cell line were all found to have elevated SET levels. Likewise, c-Myc was also overexpressed in all of the prostate cancer cells compared to the normal prostate tissue. While total PP2Ac levels were consistent between the cancer and non-cancerous cell lines, PP2Ac phosphorylation at tyrosine 307 was higher in cancer cell lines indicating that the PP2A was inactive. Ceramide levels were examined in these cells, and it was found to induce apoptosis (60), a known activator of PP2A (49) and a direct inhibitor of SET (56). Therefore, endogenous ceramide levels were measured in cancerous PC-3 and LNCaP cells and compared to normal prostate epithelium. The PC-3 cells were found to have elevated C_20_-ceramide and a decrease in C_14_-, C_16_-, C_18_-, C_22_-, C_24_-, and C_26_-ceramides. The LNCaP cells had an elevated C_16_-, C_18_-, and C_20_- and lower levels of C_14_, C_22_-, C_24_-, and C_26_-ceramides.

To increase ceramide levels, synthetic C_6_-ceramide was given at concentrations ranging from 5 to 40 μM and cell viability assayed 24 and 48-h post-treatment (59). While normal prostate epithelium as unaffected at any dose or time point, all three prostate cancer cell lines showed a dose- and time-dependent cell death. Importantly, treatment with C_6_-ceramide (10 μM, 48 h) reduced c-Myc levels in each of the cancer cell lines. Moreover, pretreatment with the PP2A inhibitor okadaic acid (10 nM) increased basal c-Myc levels and prevented a decrease following treatment with C_6_-ceramide (10 μM, 48 h).

Recently, Chen et al. found that treating AML-ETO^+^ cell lines with FTY720-induced caspase-depended apoptosis through a PP2A-dependent pathway (61). To unravel the mechanisms involved in FTY720-induced cell death in these cells, the authors performed gene microarray analyses. Among the genes found to be upregulated by FTY720 were acid sphingomyelinase, acid beta-glucosidase and sphingolipid delta(4)-desaturase, all of which are involved in the regulation of ceramide metabolism. Further study using qPCR found another ceramide-generating gene, neutral sphingomyelinase 2 (nSmase2) to be upregulated with FTY720 treatment. Accordingly, using high-performance liquid chromatography–electrospray ionization tandem mass spectrometry (HPLC-ESI-MS/MS), the authors found an increase in ceramide levels in FTY720-treated Kasumi-1 cells. In particular, C_18_-, C_20_-, and C_22_-ceramides were increased in a time (4–6 h) and dose (7.5–10 μM)-dependent manner. Particularly, these same ceramides were enriched in the mitochondrial fraction of FTY720-treated cells with C_22_-ceramide showing a dominant increase. Intriguingly, sphingosine-1-phosphate (S1P), a known inhibitor of PP2A activity (11), was found to be decreased following FTY720 treatment. Accordingly, ceramide synthase inhibitor fumonisin B1 (FB1) and the neutral sphingomyelinase inhibitor GW4869 partially protected Kasumi-1 cells from apoptosis following FTY720 treatment (61).

## Conclusion

**Figure 3:**
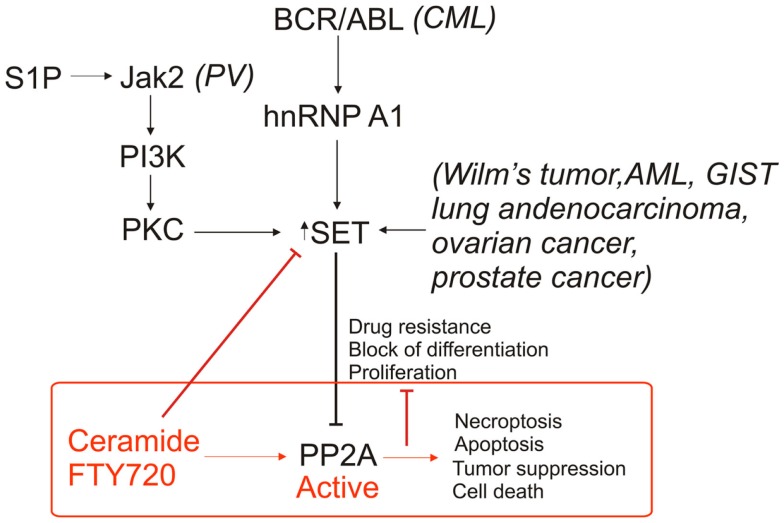
**Roles of SET/I2PP2A and ceramide/FTY720 on PP2A-dependent regulation of cell death**. PP2A inhibition is inhibited through various mechanisms in cancer via an increase in SET expression as well as altered phosphorylation of SET. Targeting SET by ceramide and/or FTY720 inhibits SET, activates PP2A, and induces cell death in various cancer cells.

Protein phosphatase 2A is commonly deregulated in cancers, typically due to inhibition by SET. The protein level of SET can be increased or SET can be subject to post-translational modification through a variety of oncogenic pathways, each resulting in the inhibition of PP2A activity (Figure 3). The commonality of PP2A inhibition by SET oncoprotein, which is overexpressed in various tumor tissues compared to non-cancerous tissue counterparts, makes SET a promising target for cancer therapeutics. Previously, most of the research on PP2A used ceramide as tool for activation. The recent data (12) provide us with the groundwork for understanding the biological properties of both ceramide/FTY720 and PP2A. Our knowledge of the relationship between sphingolipids and PP2A has been greatly enhanced through the employment of mass spectrometry, which allows for identification of specific lipids and lipid species, as well as computational modeling of SET to estimate the interactions between ceramide/FTY720 and SET. The use of sphingolipids and, synthetic sphingolipid analogs, or newly developed SET inhibitors, as cancer therapeutics is likely to increase as we are better able to target SET to reactivate PP2A, leading to tumor suppression. We believe that structural studies coupled with studies to identify the mechanism by which ceramide/SET binding regulates tumor suppressor PP2A will lead to the development of novel SET inhibitors with improved anti-cancer activity without immune suppression and other toxic effects.

## Conflict of Interest Statement

The authors declare that the research was conducted in the absence of any commercial or financial relationships that could be construed as a potential conflict of interest.
